# (1*E*)-1-[4-(Dimethyl­amino)phen­yl]pent-1-en-3-one

**DOI:** 10.1107/S1600536809046972

**Published:** 2009-11-11

**Authors:** Muhammad Nadeem Asghar, Islam Ullah Khan, Muhammad Nadeem Arshad, Jeveria Rehman

**Affiliations:** a Department of Chemistry, Forman Christian College (A Chartered University), Ferozpur Road Lahore 56400, Pakistan; b Materials Chemistry Laboratory, Department of Chemistry, GC University, Lahore 54000, Pakistan

## Abstract

The title mol­ecule, C_13_H_17_NO, is close to planar: the dihedral angle betweent the dimethyl amino group and the benzene ring is 7.94 (19)°. No significant inter­molecular inter­actions are observed in the crystal structure.

## Related literature

For background to the pharmacological effects of chalcones, see: Nielsen *et al.* (1998 ▶) and for their use as synthetic inter­mediates, see: Mukhtari *et al.* (1999 ▶). For related structures, see: Nesterov *et al.* (2007 ▶); Arshad *et al.* (2008 ▶).
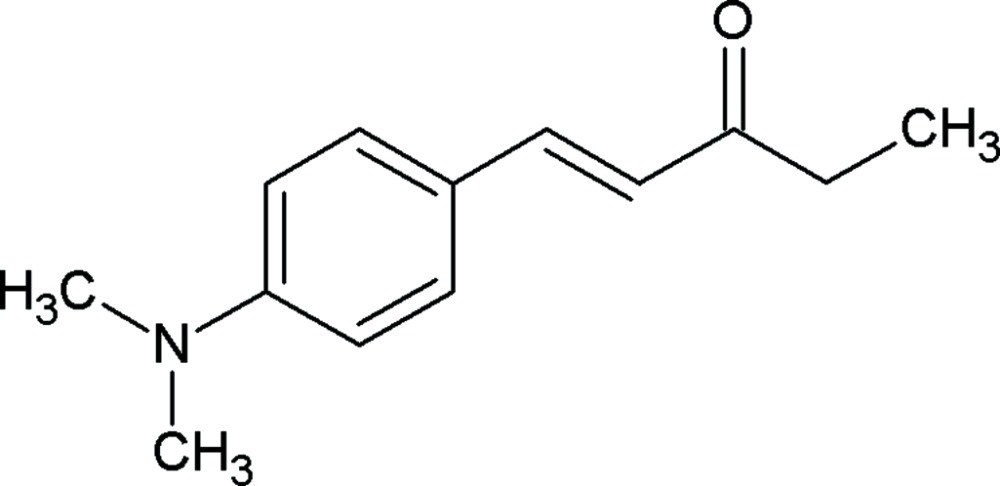


## Experimental

### 

#### Crystal data

C_13_H_17_NO
*M* *_r_* = 203.28
Monoclinic, 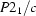

*a* = 12.6079 (14) Å
*b* = 15.1331 (17) Å
*c* = 6.2182 (6) Å
β = 100.036 (5)°
*V* = 1168.3 (2) Å^3^
*Z* = 4
Mo *K*α radiation
μ = 0.07 mm^−1^
*T* = 296 K
0.37 × 0.33 × 0.19 mm

#### Data collection

Bruker Kappa APEXII CCD diffractometer
Absorption correction: multi-scan (*SADABS*; Bruker, 2007 ▶) *T*_min_ = 0.974, *T*_max_ = 0.986
12449 measured reflections
2779 independent reflections
1051 reflections with *I* > 2σ(*I*)
*R*_int_ = 0.062

#### Refinement

*R*[*F* ^2^ > 2σ(*F* ^2^)] = 0.056
*wR*(*F* ^2^) = 0.234
*S* = 0.93
2779 reflections
139 parameters
H-atom parameters constrained
Δρ_max_ = 0.19 e Å^−3^
Δρ_min_ = −0.15 e Å^−3^

### 

Data collection: *APEX2* (Bruker, 2007 ▶); cell refinement: *SAINT* (Bruker, 2007 ▶); data reduction: *SAINT*; program(s) used to solve structure: *SHELXS97* (Sheldrick, 2008 ▶); program(s) used to refine structure: *SHELXL97* (Sheldrick, 2008 ▶); molecular graphics: *ORTEP-3* (Farrugia, 1997 ▶) and *PLATON* (Spek, 2009 ▶); software used to prepare material for publication: *WinGX* (Farrugia, 1999 ▶) and *PLATON*.

## Supplementary Material

Crystal structure: contains datablocks I, global. DOI: 10.1107/S1600536809046972/hb5213sup1.cif

Structure factors: contains datablocks I. DOI: 10.1107/S1600536809046972/hb5213Isup2.hkl

Additional supplementary materials:  crystallographic information; 3D view; checkCIF report

## Figures and Tables

**Fig. 1.**
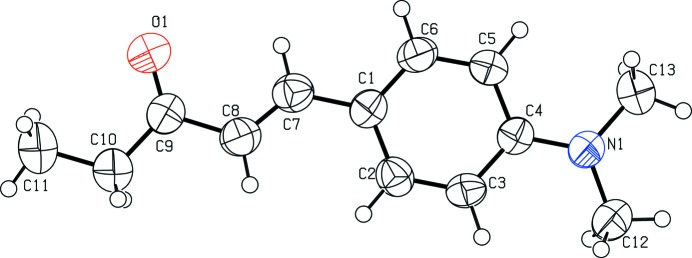
The molecular structure of (I) with displacement ellipsoids drawn at 50% probability level.

**Fig. 2.**
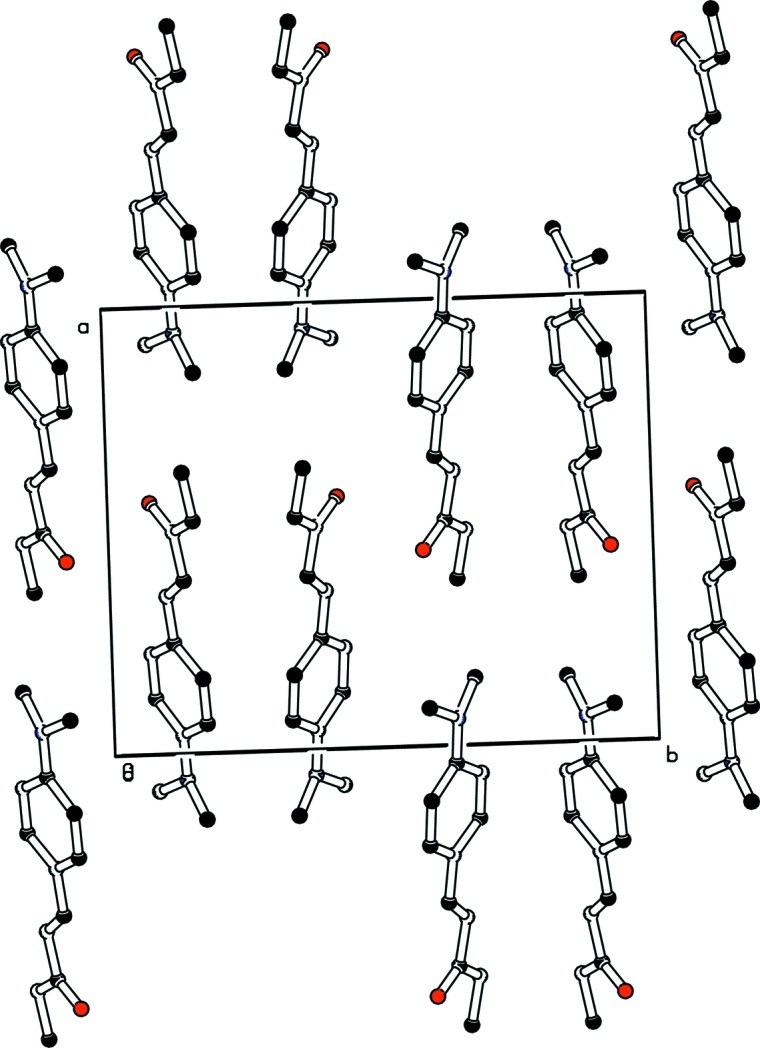
Unit cell packing diagram.

## supplementary crystallographic information

### Comment

Chalcones are biologically active compounds (e.g. Nielsen *et al.*, 1998) and have also been used as intermediates for the synthesis of 4-thiazolidinones (Mukhtari *et al.*, 1999).

The title compound (I) was synthesized by the condensation reaction of 4-(dimethylamino)benzaldehyde and 2-butanol. The molecule is structurally related to the 3,5-Bis[4-(dimethylamino)benzylidene]-1-propyl-4-piperidone(II) (Nesterov *et al.*, 2007) and (1E,4E)-1,5-Bis(4-methylphenyl)penta-1,4-dien-3-one(III) (Arshad *et al.*, 2008). The compound is almost planer while the dimethyl amino moity is oriented at dihedral angle of 7.94 (0.19) ° to the benzene ring. No significant hydrogen bonding interaction is found in the crystal structure.

### Experimental

Sodium hydroxide (0.8 g, 0.0208 mmol) was dissolved in distilled water (10 ml) and ethanol (8 ml) in a round bottom flask. The solution was cooled to room temperature. Half of the mixture of 4-(dimethylamino)benzaldehyde (1 g, 0.0083 mmol) and 2-butanol (0.60 g, 0.0083 mmol) were added to the above solution and stirred at room temperature for 15 minute then the remaining mixture was added and stirred for 2 h under the same conditions. Yellow precipitate obtained was filtered and washed with cold water. The washed precipitate was crystallized in acetone under slow evaporation to yield yellow rods of (I).

### Refinement

The H-atoms for aromatic (C—H = 0.93), methyl (C—H = 0.96) and methylene (C—H = 0.97) carbon atoms were refined geometrically and treated as riding atoms: with *U*_iso_(H) = 1.2*U*_eq_ for aromatic and methylene carbons and *U*_iso_(H) = 1.5*U*_eq_ for methyl carbon atoms.

### Crystal data

**Table:**

C_13_H_17_NO	*F*(000) = 440
*M**_r_* = 203.28	*D*_x_ = 1.156 Mg m^−^^3^
Monoclinic, *P*2_1_/*c*	Mo *K*α radiation, λ = 0.71073 Å
Hall symbol: -P 2ybc	Cell parameters from 1287 reflections
*a* = 12.6079 (14) Å	θ = 2.7–21.9°
*b* = 15.1331 (17) Å	µ = 0.07 mm^−^^1^
*c* = 6.2182 (6) Å	*T* = 296 K
β = 100.036 (5)°	Rod, yellow
*V* = 1168.3 (2) Å^3^	0.37 × 0.33 × 0.19 mm
*Z* = 4	

### Data collection

**Table:**

Bruker Kappa APEXII CCD diffractometer	2779 independent reflections
Radiation source: fine-focus sealed tube	1051 reflections with *I* > 2σ(*I*)
graphite	*R*_int_ = 0.062
φ and ω scans	θ_max_ = 27.9°, θ_min_ = 1.6°
Absorption correction: multi-scan (*SADABS*; Bruker, 2007)	*h* = −16→16
*T*_min_ = 0.974, *T*_max_ = 0.986	*k* = −19→19
12449 measured reflections	*l* = −8→8

### Refinement

**Table:**

Refinement on *F*^2^	Primary atom site location: structure-invariant direct methods
Least-squares matrix: full	Secondary atom site location: difference Fourier map
*R*[*F*^2^ > 2σ(*F*^2^)] = 0.056	Hydrogen site location: inferred from neighbouring sites
*wR*(*F*^2^) = 0.234	H-atom parameters constrained
*S* = 0.93	*w* = 1/[σ^2^(*F*_o_^2^) + (0.1169*P*)^2^] where *P* = (*F*_o_^2^ + 2*F*_c_^2^)/3
2779 reflections	(Δ/σ)_max_ < 0.001
139 parameters	Δρ_max_ = 0.19 e Å^−^^3^
0 restraints	Δρ_min_ = −0.15 e Å^−^^3^

### Special details

**Table:**

Geometry. All e.s.d.'s (except the e.s.d. in the dihedral angle between two l.s. planes) are estimated using the full covariance matrix. The cell e.s.d.'s are taken into account individually in the estimation of e.s.d.'s in distances, angles and torsion angles; correlations between e.s.d.'s in cell parameters are only used when they are defined by crystal symmetry. An approximate (isotropic) treatment of cell e.s.d.'s is used for estimating e.s.d.'s involving l.s. planes.
Refinement. Refinement of *F*^2^ against ALL reflections. The weighted *R*-factor *wR* and goodness of fit *S* are based on *F*^2^, conventional *R*-factors *R* are based on *F*, with *F* set to zero for negative *F*^2^. The threshold expression of *F*^2^ > σ(*F*^2^) is used only for calculating *R*-factors(gt) *etc*. and is not relevant to the choice of reflections for refinement. *R*-factors based on *F*^2^ are statistically about twice as large as those based on *F*, and *R*- factors based on ALL data will be even larger.

### Fractional atomic coordinates and isotropic or equivalent isotropic displacement parameters (Å^2^)

**Table:**

	*x*	*y*	*z*	*U*_iso_*/*U*_eq_	
N1	−0.05777 (18)	0.36843 (15)	1.1136 (4)	0.0547 (7)	
O1	0.5620 (2)	0.42215 (19)	0.7701 (4)	0.1083 (10)	
C1	0.2477 (2)	0.38513 (17)	0.9326 (4)	0.0519 (7)	
C2	0.1656 (2)	0.33421 (18)	0.8155 (5)	0.0563 (8)	
H2	0.1790	0.3029	0.6944	0.068*	
C3	0.0653 (2)	0.32866 (18)	0.8728 (4)	0.0529 (8)	
H3	0.0121	0.2947	0.7888	0.063*	
C4	0.0421 (2)	0.37361 (17)	1.0567 (4)	0.0472 (7)	
C5	0.1260 (2)	0.42154 (18)	1.1781 (4)	0.0539 (8)	
H5	0.1145	0.4506	1.3037	0.065*	
C6	0.2252 (2)	0.42703 (19)	1.1174 (5)	0.0577 (8)	
H6	0.2790	0.4599	1.2029	0.069*	
C7	0.3527 (2)	0.39618 (19)	0.8680 (5)	0.0601 (8)	
H7	0.4020	0.4306	0.9604	0.072*	
C8	0.3862 (2)	0.3640 (2)	0.6976 (5)	0.0628 (8)	
H8	0.3395	0.3287	0.6017	0.075*	
C9	0.4952 (3)	0.3811 (2)	0.6492 (5)	0.0633 (9)	
C10	0.5181 (3)	0.3440 (3)	0.4438 (5)	0.0832 (11)	
H10A	0.4650	0.3670	0.3252	0.100*	
H10B	0.5081	0.2805	0.4476	0.100*	
C11	0.6267 (3)	0.3614 (3)	0.3914 (6)	0.0963 (12)	
H11A	0.6404	0.4238	0.3967	0.144*	
H11B	0.6299	0.3396	0.2477	0.144*	
H11C	0.6801	0.3319	0.4960	0.144*	
C12	−0.1486 (2)	0.3348 (2)	0.9610 (5)	0.0707 (9)	
H12A	−0.1530	0.3648	0.8236	0.106*	
H12B	−0.2137	0.3447	1.0178	0.106*	
H12C	−0.1395	0.2726	0.9402	0.106*	
C13	−0.0816 (3)	0.4196 (2)	1.2966 (5)	0.0650 (9)	
H13A	−0.0345	0.4018	1.4277	0.097*	
H13B	−0.1550	0.4097	1.3127	0.097*	
H13C	−0.0711	0.4813	1.2706	0.097*	

### Atomic displacement parameters (Å^2^)

**Table:**

	*U*^11^	*U*^22^	*U*^33^	*U*^12^	*U*^13^	*U*^23^
N1	0.0483 (15)	0.0563 (15)	0.0600 (14)	−0.0042 (11)	0.0104 (11)	−0.0056 (12)
O1	0.0766 (18)	0.148 (3)	0.1051 (19)	−0.0394 (16)	0.0280 (15)	−0.0576 (18)
C1	0.0488 (17)	0.0481 (17)	0.0581 (17)	−0.0005 (13)	0.0077 (14)	0.0043 (14)
C2	0.063 (2)	0.0501 (18)	0.0582 (17)	0.0022 (15)	0.0170 (15)	−0.0019 (14)
C3	0.0561 (19)	0.0445 (17)	0.0564 (17)	−0.0048 (13)	0.0049 (14)	−0.0052 (13)
C4	0.0492 (17)	0.0371 (15)	0.0555 (16)	−0.0001 (13)	0.0094 (14)	0.0048 (13)
C5	0.057 (2)	0.0529 (18)	0.0518 (16)	−0.0017 (14)	0.0089 (14)	−0.0070 (13)
C6	0.0516 (19)	0.0588 (19)	0.0598 (18)	−0.0061 (14)	0.0015 (14)	−0.0066 (15)
C7	0.058 (2)	0.058 (2)	0.0621 (18)	0.0016 (15)	0.0047 (15)	−0.0015 (15)
C8	0.058 (2)	0.064 (2)	0.0653 (19)	−0.0031 (16)	0.0067 (16)	−0.0058 (16)
C9	0.054 (2)	0.068 (2)	0.0689 (19)	−0.0046 (16)	0.0129 (16)	−0.0040 (16)
C10	0.064 (2)	0.111 (3)	0.077 (2)	−0.007 (2)	0.0190 (17)	−0.019 (2)
C11	0.072 (2)	0.125 (3)	0.099 (3)	0.000 (2)	0.034 (2)	−0.017 (2)
C12	0.056 (2)	0.075 (2)	0.082 (2)	−0.0110 (17)	0.0134 (17)	−0.0093 (18)
C13	0.067 (2)	0.066 (2)	0.0651 (19)	0.0049 (16)	0.0215 (15)	0.0024 (16)

### Geometric parameters (Å, °)

**Table:**

N1—C4	1.368 (3)	C7—H7	0.9300
N1—C12	1.447 (3)	C8—C9	1.480 (4)
N1—C13	1.451 (4)	C8—H8	0.9300
O1—C9	1.200 (3)	C9—C10	1.469 (4)
C1—C6	1.385 (4)	C10—C11	1.485 (4)
C1—C2	1.390 (4)	C10—H10A	0.9700
C1—C7	1.458 (4)	C10—H10B	0.9700
C2—C3	1.375 (4)	C11—H11A	0.9600
C2—H2	0.9300	C11—H11B	0.9600
C3—C4	1.404 (3)	C11—H11C	0.9600
C3—H3	0.9300	C12—H12A	0.9600
C4—C5	1.391 (4)	C12—H12B	0.9600
C5—C6	1.370 (4)	C12—H12C	0.9600
C5—H5	0.9300	C13—H13A	0.9600
C6—H6	0.9300	C13—H13B	0.9600
C7—C8	1.302 (4)	C13—H13C	0.9600
			
C4—N1—C12	120.6 (2)	O1—C9—C10	121.3 (3)
C4—N1—C13	119.9 (2)	O1—C9—C8	122.6 (3)
C12—N1—C13	117.0 (2)	C10—C9—C8	116.1 (3)
C6—C1—C2	116.5 (3)	C9—C10—C11	116.9 (3)
C6—C1—C7	120.2 (3)	C9—C10—H10A	108.1
C2—C1—C7	123.2 (3)	C11—C10—H10A	108.1
C3—C2—C1	122.2 (3)	C9—C10—H10B	108.1
C3—C2—H2	118.9	C11—C10—H10B	108.1
C1—C2—H2	118.9	H10A—C10—H10B	107.3
C2—C3—C4	120.8 (3)	C10—C11—H11A	109.5
C2—C3—H3	119.6	C10—C11—H11B	109.5
C4—C3—H3	119.6	H11A—C11—H11B	109.5
N1—C4—C5	122.4 (2)	C10—C11—H11C	109.5
N1—C4—C3	120.9 (3)	H11A—C11—H11C	109.5
C5—C4—C3	116.6 (3)	H11B—C11—H11C	109.5
C6—C5—C4	121.7 (3)	N1—C12—H12A	109.5
C6—C5—H5	119.1	N1—C12—H12B	109.5
C4—C5—H5	119.1	H12A—C12—H12B	109.5
C5—C6—C1	122.0 (3)	N1—C12—H12C	109.5
C5—C6—H6	119.0	H12A—C12—H12C	109.5
C1—C6—H6	119.0	H12B—C12—H12C	109.5
C8—C7—C1	128.4 (3)	N1—C13—H13A	109.5
C8—C7—H7	115.8	N1—C13—H13B	109.5
C1—C7—H7	115.8	H13A—C13—H13B	109.5
C7—C8—C9	123.0 (3)	N1—C13—H13C	109.5
C7—C8—H8	118.5	H13A—C13—H13C	109.5
C9—C8—H8	118.5	H13B—C13—H13C	109.5
			
C6—C1—C2—C3	3.0 (4)	C4—C5—C6—C1	−0.2 (4)
C7—C1—C2—C3	−176.3 (3)	C2—C1—C6—C5	−2.4 (4)
C1—C2—C3—C4	−1.1 (4)	C7—C1—C6—C5	177.0 (3)
C12—N1—C4—C5	166.5 (3)	C6—C1—C7—C8	−177.9 (3)
C13—N1—C4—C5	4.9 (4)	C2—C1—C7—C8	1.4 (5)
C12—N1—C4—C3	−14.8 (4)	C1—C7—C8—C9	179.4 (3)
C13—N1—C4—C3	−176.4 (2)	C7—C8—C9—O1	3.8 (5)
C2—C3—C4—N1	179.8 (3)	C7—C8—C9—C10	−176.6 (3)
C2—C3—C4—C5	−1.4 (4)	O1—C9—C10—C11	−1.2 (5)
N1—C4—C5—C6	−179.1 (2)	C8—C9—C10—C11	179.3 (3)
C3—C4—C5—C6	2.1 (4)		
